# The Gut Microbiota of Newborn Calves and Influence of Potential Probiotics on Reducing Diarrheic Disease by Inhibition of Pathogen Colonization

**DOI:** 10.3389/fmicb.2021.772863

**Published:** 2021-10-21

**Authors:** Peixin Fan, Miju Kim, Grace Liu, Yuting Zhai, Ting Liu, Joseph Danny Driver, Kwangcheol C. Jeong

**Affiliations:** ^1^Emerging Pathogens Institute, University of Florida, Gainesville, FL, United States; ^2^Department of Animal Sciences, University of Florida, Gainesville, FL, United States

**Keywords:** gut microbiota, diarrhea, newborn calves, *Lactobacillus*, probiotics

## Abstract

Calf diarrhea is one of the most concerning challenges facing both the dairy and beef cattle industry. Maintaining healthy gut microbiota is essential for preventing gastrointestinal disorders. Here, we observed significantly less bacterial richness in the abnormal feces with watery or hemorrhagic morphology compared to the normal solid feces. The normal solid feces showed high relative abundances of *Osllospiraceae*, *Christensenellaceae*, *Barnesiella*, and *Lactobacillus*, while the abnormal feces contained more bacterial taxa of *Negativicutes*, *Tyzzerella*, *Parasutterella*, *Veillonella*, *Fusobacterium*, and *Campylobacter*. Healthy calves had extensive bacterial-bacterial correlations, with negative correlation between *Lactobacillus* and potential diarrheagenic *Escherichia coli-Shigella*, but not in the abnormal feces. We isolated *Lactobacillus* species (*L. reuteri*, *L. johnsonii*, *L. amylovorus*, and *L. animalis*), with *L. reuteri* being the most abundant, from the healthy gut microbiota. Isolated *Lactobacillus* strains inhibited pathogenic strains including *E. coli* K88 and *Salmonella* Typhimurium. These findings indicate the importance of a diverse gut microbiota in newborn calf’s health and provide multiple potential probiotics that suppress pathogen colonization in the gastrointestinal tract to prevent calf diarrhea.

## Introduction

Newborn calves are susceptible to diarrhea, especially during the first month of their life (Bendali et al., 1999). The US Department of Agriculture estimated that between 4 and 25% of calves die from diarrhea each year in the US, leading to tremendous economic losses in the cattle industry (USDA, 2017). Calf diarrhea is challenging to mitigate because of its multifactorial etiology, which comprises of both infectious and noninfectious factors (Whon et al., 2021). Antibiotics are commonly used as a treatment for calf diarrhea, but the efficacy of antibiotic treatment is questionable (Kim et al., 2021). Furthermore, antibiotic use is nontargeted that can negatively impact calf gut microbial composition (Ianiro et al., 2016; Ramirez et al., 2020).

A healthy gut microbiota prevent colonization of foreign pathogens (Pickard et al., 2017; Fan et al., 2019, 2020, 2021; Kim et al., 2021) and enhance host immune systems through interactions between antigens and immune cells during early stage of life (Zhao and Elson, 2018; Fan et al., 2020). The increased intestinal permeability and disturbance of gut microbiota are key factors leading to the pathogen-induced diarrhea. The association of calf diarrhea with both microbial composition and functional dysbiosis shed light to ameliorate diarrhea through modulating gut microbial ecosystems. A study attempted to perform fecal microbiota transplantation (FMT) from healthy donors to six Korean brown cattle calves exhibiting diarrhea and showed promising results in which incidence of diarrhea was reduced significantly (Kim et al., 2021). In addition, administration of oral probiotics can be possible preventative treatments or supportive therapies for calf diarrhea. Duration of calf diarrhea has been shown to be diminished through administration of multispecies probiotics and yeast bolus (Renaud et al., 2019). To develop more efficient probiotics for diarrhea mitigation strategies, it is necessary to characterize the gut microbiota in healthy and diarrheic calves. A few studies have investigated the difference in gut microbial composition between healthy and diarrheic dairy calves and indicated the critical role of microbiota development in regulating gut health during early stage (Gomez et al., 2017; Hennessy et al., 2021). However, the important gut commensals that contribute to preventing diarrhea in newborn beef cattle, especially raised on pasture remain poorly defined.

In this study, we seek to elucidate the role of gut microbiota in the progression of diarrhea in one-month-old calves and identified several potential probiotics that may contribute to the gut health of young calves through suppressing diarrheagenic bacteria. The findings will aid in the future development of dietary interventions that may resolve or prevent enteric and diarrheal disease in young calves.

## Materials and Methods

### Animal Management

Preweaning calves enrolled in this study were from one generation of the multibreed Angus-Brahman (MAB) herd of the University of Florida. The calves were naturally born on pasture during the delivery season. The birthweight of newborns was measured within 24h after delivery. The calves were raised together with dams during preweaning stage on pasture on the cow-calf operation in Santa Fe River Ranch Beef Unit, FL, United States.

### Fecal Sample Collection, Morphology Categorization, and Sample Processing

Fecal samples were collected from 91 beef calves ranging in age from 21 to 35days. Briefly, each fecal sample was collected from the rectal-anal junction (RAJ) using two sterile cotton swabs. During sample collection, the morphology of fecal samples was observed and recorded based on criteria of Bristol stool scale, especially the color, consistency, and presence of blood (Koppen et al., 2016). Swabs with fecal samples were placed in a 15ml conical tube on ice and were transported to the laboratory within 1h for further processing. Each swab sample was resuspended in mixture of 2ml of Luria-Bertani (LB) broth and 2ml of 30% glycerol as previously described (Fan et al., 2020). The fecal solution was then split into four 2ml tubes and frozen in an ultra-low freezer at −80°C.

### 16S rRNA Gene Amplicon Sequencing

Fecal samples were thawed on ice and homogenized. Then 1ml of each sample was used for DNA extraction using the QIAamp PowerFecal DNA kit according to the manufacturer’s instructions (Qiagen, United States). To understand the bacterial community, a dual-index sequencing strategy was applied (Kozich et al., 2013). Briefly, the V4 region of the 16S rRNA gene was amplified using the PCR with dual-index primers (Kozich et al., 2013). The PCR amplification reaction included 1μl forward index primer (10mM), 1μl reverse index primer (10mM), 1μl 10ng/μl DNA template, and 17μl Pfx AccuPrime master mix (Invitrogen, United States). Amplification was initiated with denaturation for 5min at 95°C, followed by 30cycles of 95°C for 30s, annealing at 55°C for 30s and extension at 72°C for 1min, with a final elongation for 5min at 72°C. The amplicons were then purified and normalized using the SequalPrep plate normalization kit (Invitrogen, United States). The same amount of barcoded V4 amplicons from each sample was pooled to construct the DNA library.

### Analysis for 16S rRNA Gene Amplicon Sequencing

The sequencing data of 16S rRNA gene were analyzed using version 2 of the Quantitative Insights into Microbial Ecology (QIIME 2) pipeline (Bolyen et al., 2019). Briefly, paired-end raw reads were imported, and the quality of the initial bases was evaluated according to the Interactive Quality Plot. The sequence quality control was performed with the Divisive Amplicon Denoising Algorithm (DADA2) pipeline implemented in QIIME 2, including steps for filtering low quality reads, denoising reads, merging the paired-end reads, and removing chimeric reads. The phylogenetic tree was generated using the align-to-tree-mafft-fasttree pipeline from the q2-phylogeny plugin of QIIME 2. The sequencing depth was normalized to 10,800 sequences per sample. The number of amplicon sequence variants (ASVs), Shannon index, and Bray-Curtis distance were calculated by the core-metrics-phylogenetic method. All ASVs were classified into the bacterial taxonomy using the q2-feature-classifier plugin of QIIME 2 and the SILVA 138 database.^1^

### Co-occurrence Network Analysis

To investigate bacterial-bacterial interactions in the gut bacterial community of one-month-old calves, co-occurrence events of core bacterial genera that were present in at least 50% of normal and abnormal fecal samples were evaluated in the network interface, respectively, using pairwise Spearman’s rank correlations (*r_s_*) based on the relative abundance according to the previous study (Fan et al., 2020). The Spearman rank correlation was analyzed using Hmisc within RStudio (version 1.1456). A significant rank correlation between two bacterial genera (*r_s_*>0.2 or *r_s_*<−0.2, FDR-adjusted *p* value<0.05) was considered as a co-occurrence event. The network was visualized using the Force Atlas algorithm in the interactive platform Gephi 0.9.2.^2^ In the network, nodes represented different genera, and edges indicated significant correlations between nodes. The size of the nodes represented the degree of connection, and the thickness of edges indicated the strength of the correlation.

### Microbial Function Prediction

Functional capacity of the gut microbial community was predicted using the online Galaxy version of phylogenetic investigation of communities by reconstruction of unobserved states (PICRUSt; http://galaxy.morganlangille.com/; Langille et al., 2013). The closed reference operational taxonomic unit (OTU) table was generated by picking OTU against the 13 August 2013 Greengenes database using QIIME (version 1.9.0). Normalization of copy numbers, metagenome prediction, and function categorization based on Kyoto Encyclopedia of Genes and Genomes (KEGG) pathways were conducted according to a standard analysis process.

### Isolation of *Lactobacillus* spp. Strains and Antimicrobial Activities

The normal and abnormal feces were plated after 10-fold serial dilution in phosphate-buffered saline (PBS) on de Man, Rogosa, and Sharpe (MRS) agar (Difco, United States) to determine the concentrations of lactic acid bacteria (LAB), then plates were anaerobically incubated at 37°C for 48h using the GasPak EZ Anaerobe System (BD, United States). To isolate *Lactobacillus* strains from the normal feces, colonies with different morphologies were randomly selected and purified on the same solid media to isolate pure colonies. A total of 79 colonies were isolated from fecal samples of healthy calves. Genomic DNA of the isolated colonies was extracted by bead beating method, as previously described (Ma et al., 2021). Then, to speciate the isolates, the 16S gene was amplified using the universal primer pair KCP812 (5'-CAG GCC TAA CAC ATG CAA GTC-3') and KCP813 (5'-GGG CGG WGT GTA CAA GGC-3'; Marchesi et al., 1998). Amplified PCR products were purified using the QIAquick PCR purification kit (Qiagen, United States). Purified PCR products were sequenced in Genewiz (South Plainfield, United States), and the 16S rRNA gene sequences were identified by BLAST searching against National Center for Biotechnology Information (NCBI) 16S ribosomal RNA sequences database.

Antimicrobial activity of the *Lactobacillus* isolates was assessed against *Escherichia coli* KCJ2K2616, *E. coli* K88 KCJ4567, and *Salmonella enterica* Typhimurium KCJ187. For antimicrobial activity of the isolates, the agar well diffusion method was used with slight modification (Espeche et al., 2009; Boranbayeva et al., 2020). Briefly, antimicrobial activities of filter (0.22μm) sterilized supernatants of 18h cultures of *Lactobacillus* spp. were tested against pathogens described above. Overnight cultures of pathogens (10^7^CFU) were mixed with soft LB agar medium (0.75% agar) and solidified. Then, 100μl of the supernatants was loaded in the agar well. After overnight incubation at 37°C, the diameters of inhibition zones were measured. *Lactoplantibacillus plantarum* (formerly known as *Lactobacillus plantarum*) KCJ4051 was used as a positive control (Yu et al., 2021), and each strain was tested in triplicate.

### Statistical Analysis

Differences in alpha diversity and antimicrobial activity were analyzed using Student’s *t*-test or a one-way ANOVA followed by an F-test and Tukey’s HSD test for pairwise comparison of multiple means using the GraphPad prism. Differences between Bray-Curtis distances were analyzed using a permutational multivariate ANOVA (PERMANOVA) with the beta-group-significance command in QIIME 2 pipeline.

A Linear discriminant analysis Effect Size (LEfSe) analysis was applied to identify unique bacteria in normal and abnormal feces based on the non-parametric factorial Kruskal–Wallis sum-rank test. An effect size of 2 was considered as significant difference.

## Results

### High Prevalence of Abnormal Feces in One-Month-Old Beef Calves

**Figure 1:**
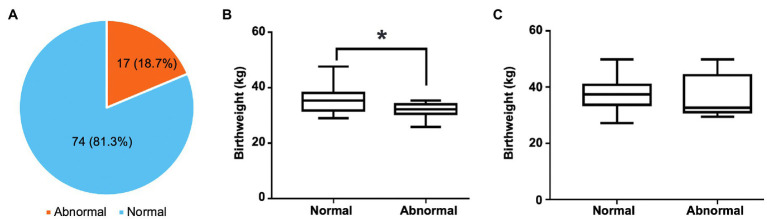
The distribution of normal and abnormal feces and its relationship with birthweight of preweaning calves. **(A)** The prevalence of normal and abnormal feces of preweaning calves. **(B)** The difference in birthweight between heifers with normal feces and those with abnormal feces. **(C)** The difference in birthweight between bulls with normal feces and those with abnormal feces. In the box plot, the asterisk reflects that they are significantly different (*p*<0.05) based on the Student’s *t*-test.

One-month-old calves had high prevalence of abnormal feces. Among the 91 fecal samples, 74 (81.3%) were firm and brownish, which were classified as normal feces, whereas 17 (18.7%) were abnormal, including six mild watery, two extremely watery, eight with slight blood observed, and one extremely hemorrhagic (Figure 1A; Supplementary Table 1). Twelve out of 17 abnormal feces were from heifer. Interestingly, the birthweight of heifer with abnormal feces were significantly lower than those with normal feces (31.86±2.69kg vs. 35.26±4.50kg, *p*=0.0178, Figure 1B), suggesting birth weight might be associated with gut health. However, the pattern was not observed in bulls (38.44±8.13kg vs. 38.14±5.17kg; Figure 1C).

### Normal and Abnormal Feces in Young Calves Carried Distinctive Microbiota

To compare the microbiota composition of normal and abnormal feces, the sequencing of the V4 region of the 16S gene was conducted. A total of 2,172,004 (23,868±455) raw reads were generated and after removing substitution and chimera errors using DADA2 pipeline, 1,703,949 (18,724±367) high-quality ASVs were yielded (Supplementary Table 2).

**Figure 2:**
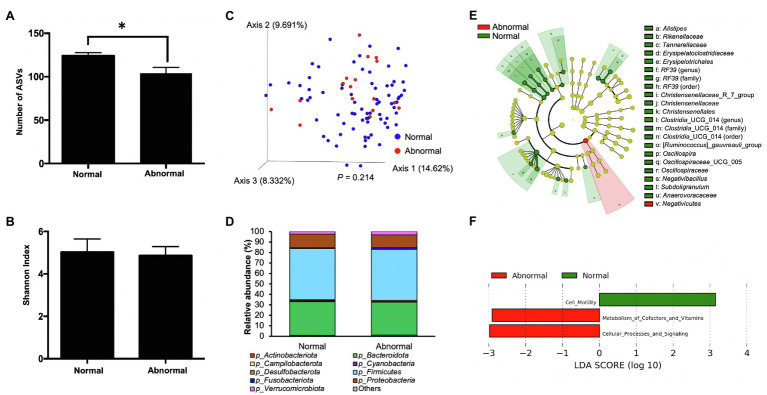
The microbiota characteristics of normal and abnormal feces. **(A)** The number of amplicon sequence variants (ASVs) of normal and abnormal feces. **(B)** The Shannon index of normal and abnormal feces. **(C)** The PCoA plot based on Bray-Curtis distances comparing microbiota composition between normal and abnormal feces. The difference in Bray-Curtis distance was analyzed by the permutational multivariate ANOVA (PERMANOVA). **(D)** Distribution of abundant bacterial phyla in normal and abnormal feces. **(E)** The taxonomic cladogram obtained from the linear discriminant analysis effect size (LEfSe) analysis showing the unique bacterial taxa in the normal and abnormal feces. **(F)** Results of the LEfSe analysis showing microbial function that were significantly different between normal and abnormal feces.

Alpha diversity analysis was first conducted to compare the richness and diversity of bacterial community between the normal and abnormal feces using the alpha diversity metrics, the number of ASVs, and the Shannon index, respectively. The abnormal feces had significantly lower number of ASVs compared with normal feces (*p*=0.017, Figure 2A), indicating a lower gut bacterial richness in calves with abnormal feces. However, we did not observe an apparent difference in the Shannon Index between groups (*p*=0.2841, Figure 2B), denoting comparable levels of species diversity. Moreover, visualization of the differences in gut microbiota structure between the abnormal and normal fecal groups *via* a principal coordinates analysis (PCoA) plot showed no significant difference (*p*=0.214) between groups based on Bray-Curtis distance evaluated using PERMANOVA (Figure 2C). Both feces were dominated by *Firmicutes*, *Bacteroidota*, and *Proteobacteria* phyla (Figure 2D; Supplementary Table 3). To differentiate significant bacterial taxa between the fecal groups, a cladogram was generated based on LefSe. The abnormal feces harbored higher relative abundance of *Negativicutes*, while normal feces had higher relative abundance of commensals, including *Rikenellaceae*, *Tannerellaceae*, *Erysipelatoclostridiaceae*, *Christensenellaceae*, *Oscillospiraceae*, and *Anaerovoraceae* families (Figure 2E). To understand the influence of different microbiota on microbial functional properties, the PICRUSt was applied to predict the relative abundance of bacterial genes. The bacterial genes involved in metabolism of cofactors and vitamins, and cellular processes and signaling were enriched in abnormal feces, whereas those participate in cell motility had higher relative abundance in normal feces (Figure 2F).

### The Variation in Microbiota of Abnormal Feces With Different Morphologies

**Figure 3:**
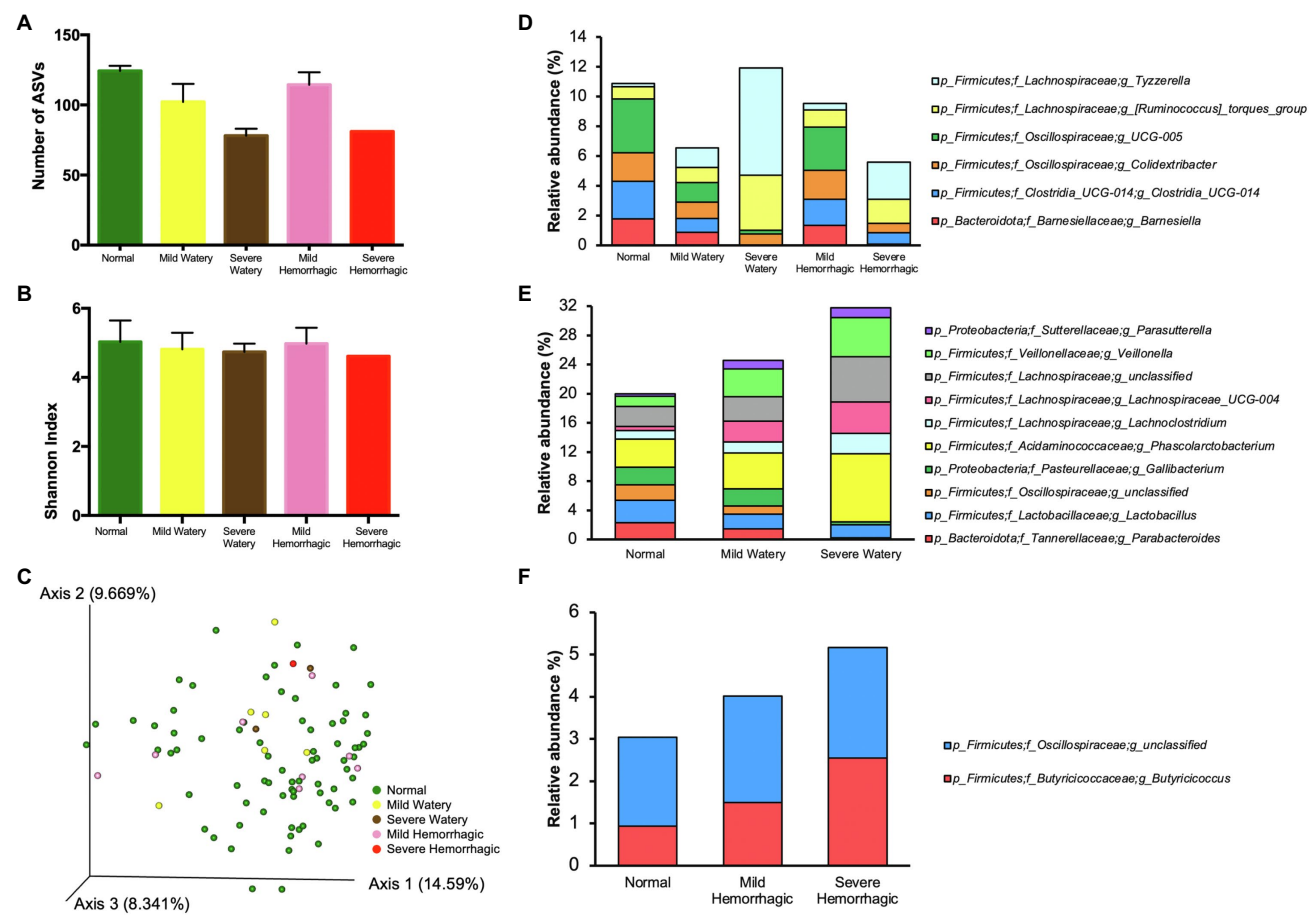
The microbiota characteristics of feces with different morphologies. **(A)** The number of ASVs of feces with different morphologies. **(B)** The Shannon index of feces with different morphologies. **(C)** The PCoA plot based on Bray-Curtis distances comparing microbiota composition between feces with different morphologies. **(D)** The bacterial genera that either showed highest relative abundance or lowest relative abundance in normal feces compared with other morphologies. **(E)** The bacterial genera that were gradually changed with the severity of fecal watery morphology. **(F)** The bacterial genera that were gradually changed with the severity of hemorrhagic morphology.

After comparing gut microbiota composition between calves with abnormal feces and calves with normal feces, the abnormal fecal group was subcategorized based on differing morphologies (mild watery, severe watery, mild hemorrhagic, and severe hemorrhagic) for further analysis. The number of ASVs was lower in watery feces and was dependent on severity of the watery property (Figure 3A). Average number of ASVs for normal, mild watery, and severe watery feces was 124.3, 102.2, and 78.00, respectively. The feces with presence of slight blood contained slightly lower level of ASVs (114.4) compared to normal feces, but the severe hemorrhagic feces harbored much fewer ASVs (81.0). Similar but less obvious pattern was observed for the Shannon index (Figure 3B). The results show an incremental decrease in bacterial species richness and diversity with increased levels of severity of both hemorrhagic and watery feces. However, no significant difference was observed for overall microbiota structure between severe watery feces and severe hemorrhagic feces with the normal feces according to the PCoA plot based on Bray-Curtis distance (Figure 3C).

We further pinpointed key differences in microbiota composition of feces with varied morphologies. The microbiota at phylum level was similar among normal feces, mild watery feces, and mild hemorrhagic feces, while severe watery feces contained 20 folds more *Fusobacteriota* compared with normal feces, and severe hemorrhagic feces had much less level of *Verrucomicrobiota* (Supplementary Figure 1). At genus level, normal feces harbored highest relative abundance of *Barnesiella* and *Clostridia*_UCG-014, *Colidextribacter* and *Oscillospiraceae_*UCG_005 but lowest relative abundance of [*Ruminococcus*]_*torques*_group and *Tyzzerella* compared to feces with other morphologies (Figure 3D; Supplementary Table 4). Moreover, the relative abundances of *Parabacteroides*, *Lactobacillus*, unclassified *Oscillospiraceae*, and *Gallibacterium* were gradually decreased with the increase of severity of the watery morphology, whereas the relative abundances of *Phascolarctobacterium*, *Lachnoclostridium*, *Lachnospiraceae*_UCG-004, *Veillonella*, and *Parasutterella* were elevated, indicating these bacteria were sensitive to fecal watery morphology (Figure 3E; Supplementary Table 5). For the hemorrhagic morphology, *Butyricicoccus* and unclassified *Oscillospiraceae* were gradually enriched in feces with increase of severity (Figure 3F; Supplementary Table 6).

### The Microbiota Profile of Severe Watery and Hemorrhagic Feces

**Figure 4:**
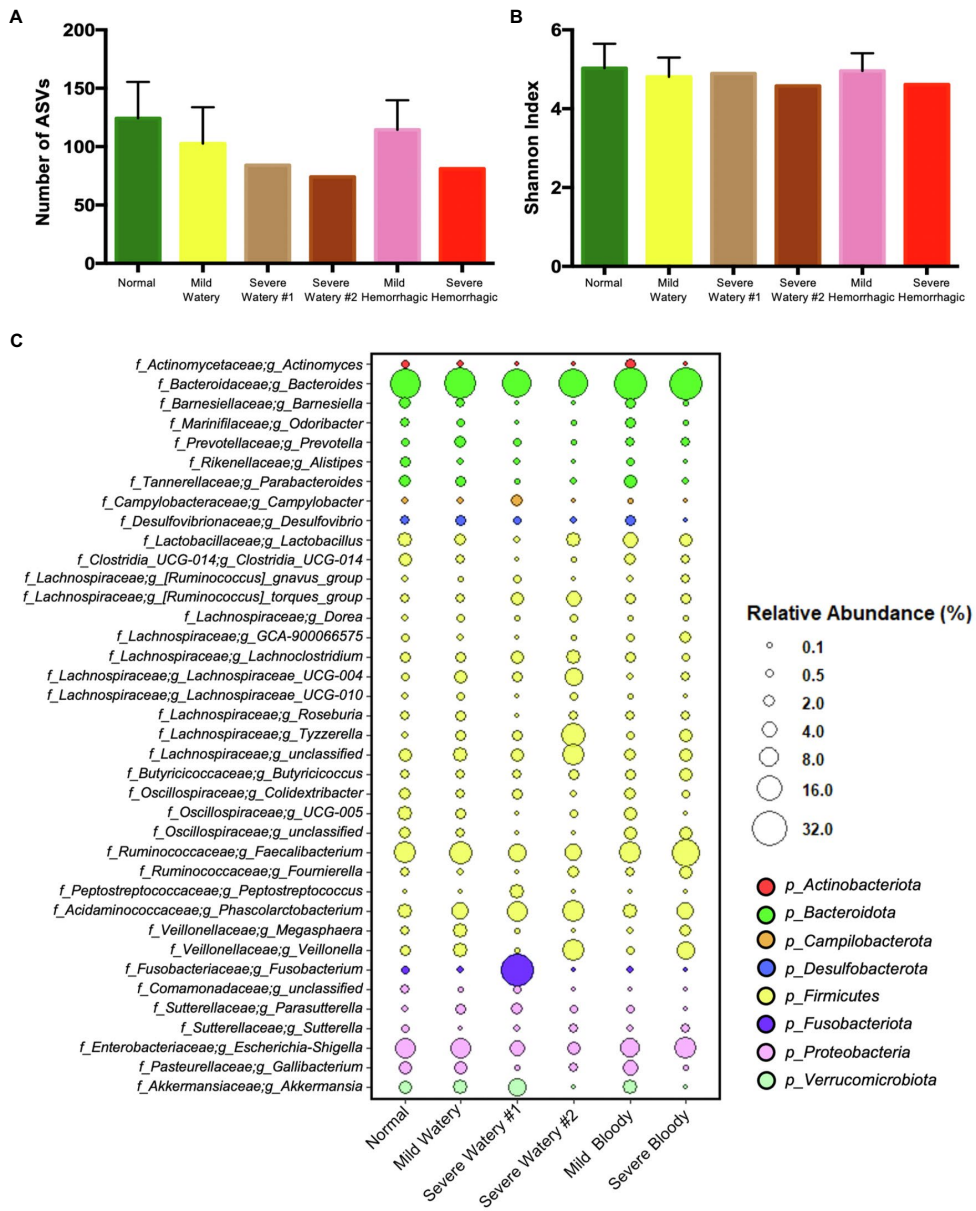
The microbiota characteristics of severe watery or hemorrhagic feces. **(A)** The number of ASVs of subgroup feces. **(B)** The Shannon index of subgroup feces. **(C)** The dotplot that shows the relative abundance of genera in subgroup feces.

We further extracted the individual severe watery and hemorrhagic fecal samples and characterized their microbiota structure, as they would pose greater risk to the intestinal health of calves. All severe abnormal fecal samples had much less bacterial richness according to the number of ASVs (Figure 4A) and slightly lower bacterial diversity according to the Shannon index (Figure 4B). For the microbiota composition, one of the severe watery feces (Severe Watery #1) harbored more than 5-fold higher relative abundance of *Fusobacterium* (26.56%), *Peptostreptococcus* (3.23%), *Campylobacter* (2.96%), *Parasutterella* (2.25%), and *Tyzzerella* (1.18%) compared to normal feces (0.61, 0.01, 0.34, 0.36, and 0.22%), and the other one (Severe Watery #2) contained more *Tyzzerella* (13.27%), *Veillonella* (10.52%), *Lachnospiraceae*_UCG-004 (6.94%), [*Ruminococcus*]_*torques*_group (4.53%) than normal feces (0.22, 1.37, 0.57, and 0.8%; Figure 4C). For the severe hemorrhagic feces, it shed more than 10-fold higher relative abundance of *Tyzzerella* (2.5%) and approximately 5-fold more *Veillonella* (6.06%) than normal feces (0.22 and 1.37%; Figure 4C).

### Bacterial-Bacterial Interactions Predicted by Co-occurrence Network

**Figure 5:**
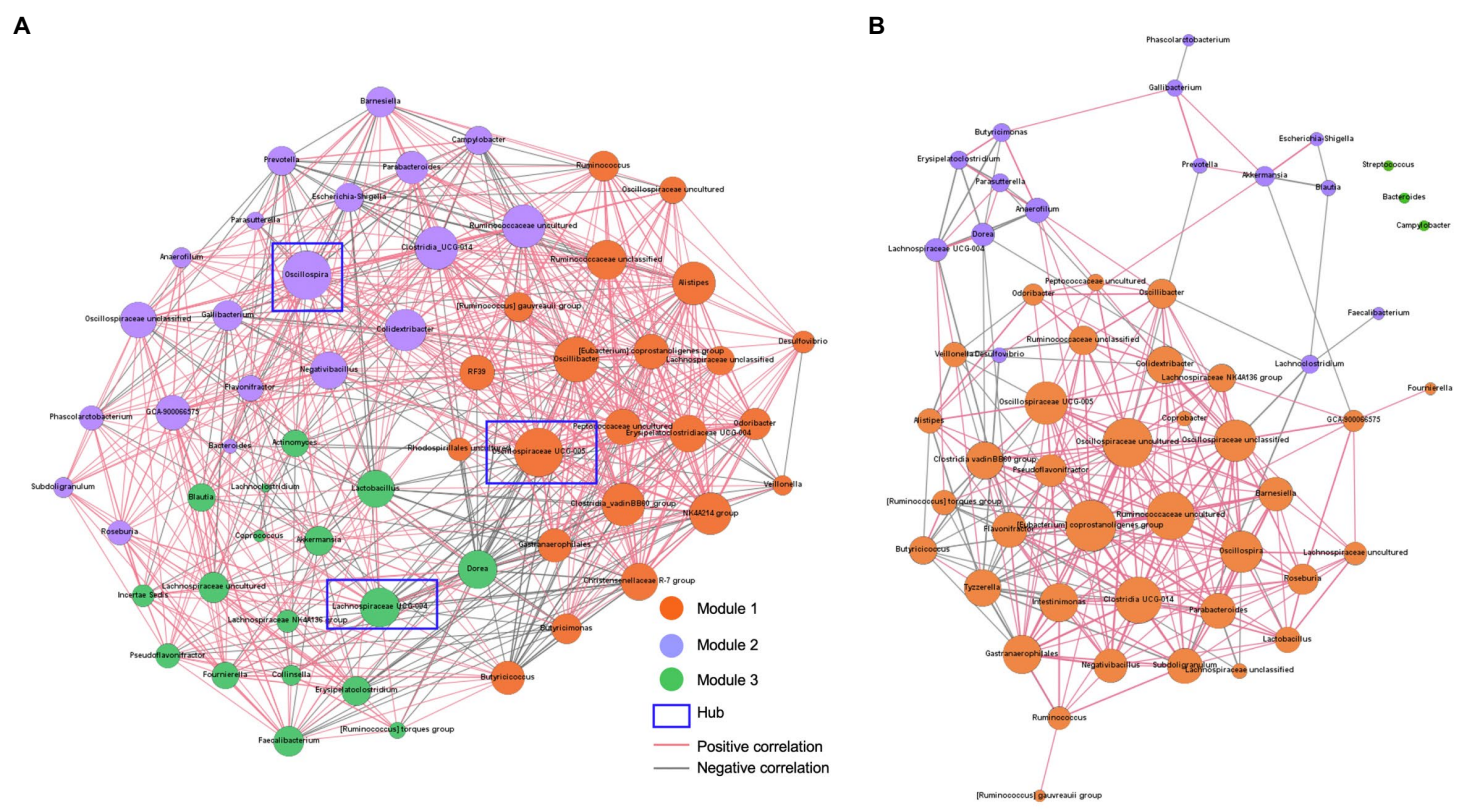
Bacterial-bacterial interactions predicted by co-occurrence networks. **(A)** Bacterial co-occurrence network in normal feces. **(B)** Bacterial co-occurrence network in abnormal feces. Co-occurrence networks predicting the bacteria-bacteria interactions among core bacterial genera (present in more than 50% of normal or abnormal fecal samples) in calf feces. Connections were detected based on Spearman’s rank correlations (*r_s_*>0.2 or *r_s_*<−0.2, FDR adjusted *p* value<0.05). Dot sizes represent number of connections with other taxa. Thickness of lines represents strength of relatedness. The modules were designated based on a community detection algorithm.

To investigate whether there are differences in bacterial-bacterial interactions in the gut microbial ecosystems, the co-occurrence networks were analyzed using the 59 and 54 core bacterial genera that were present in more than 50% of the normal and abnormal fecal samples, respectively. The normal feces had eight unique core bacterial genera including *Actinomyces*, *Christensenellaceae* R-7 group, *Collinsella*, *Coprococcus*, *Erysipelatoclostridiaceae* UCG-004, *Incertae Sedis*, *Oscillospiraceae* NK4A214 group, RF39, and *Rhodospirillales* uncultured, whereas the abnormal feces had only four unique core bacterial genera including *Coprobacter*, *Intestinimonas*, *Streptococcus*, and *Tyzzerella*. The normal feces had more bacteria-bacteria interactions (*n*=656) compared to the abnormal feces (*n*=272) based on Spearman correlations (*P*adjust<0.05, *r_s_*>0.2, or *r_s_*<−0.2). For the normal feces, the network was grouped into three modules according to the inner interactions among the nodes (Figure 5A). In module 1, *Oscillospira*, which had higher relative abundance in normal feces (Figure 2E), was the hub among the 22 core bacterial genera, having 38 significant correlations with other bacteria (Figure 5A). Notably, *Oscillospira* was negatively associated with *Campylobacter*, which showed high relative abundance in the severe watery feces, and *Escherichia-Shigella*, which contain multiple pathogenic species that can cause diarrhea in young calves. In module 2, *Oscillospiraceae UCG-005* was the hub among the 20 core bacteria, having 38 correlations with other bacteria. *Oscillospiraceae* UCG-005 showed negative associations with *Veillonella*, which was enriched in watery feces (Figure 3E), as well as *Campylobacter* in Module 1. In module 3, *Lachnospiraceae* UCG-004 associated with watery feces was the hub among the 17 core bacteria and it was negatively correlated with the *Christensenellaceae* R-7 group in module 1, which was belonged to the unique core bacteria of the normal feces. Importantly, a significant negative correlation was also observed between beneficial bacteria *Lactobacillus* and *Escherichia-Shigella*.

For the abnormal feces, the bacterial-bacterial interaction network was also grouped to three modules, with module 1 being the dominant, but the number of nods and correlations were significantly less compared to normal feces (Figure 5B). The [*Eubacterium*] *coprostanoligenes* group was the hub among the 38 core bacteria, with 26 significant correlations with other bacteria. Notably, the negative associations between potential beneficial bacteria, *Oscillospira* and *Lactobacillus*, and opportunistic pathogens, *Campylobacter* and *Escherichia-Shigella*, which were detected in the normal feces, were not observed in the abnormal feces, suggesting that beneficial bacteria may suppress diarrheagenic pathogens in normal healthy gut microbiota.

### Identification of *Lactobacillus* spp. Strains and Their Antimicrobial Activities

**Figure 6:**
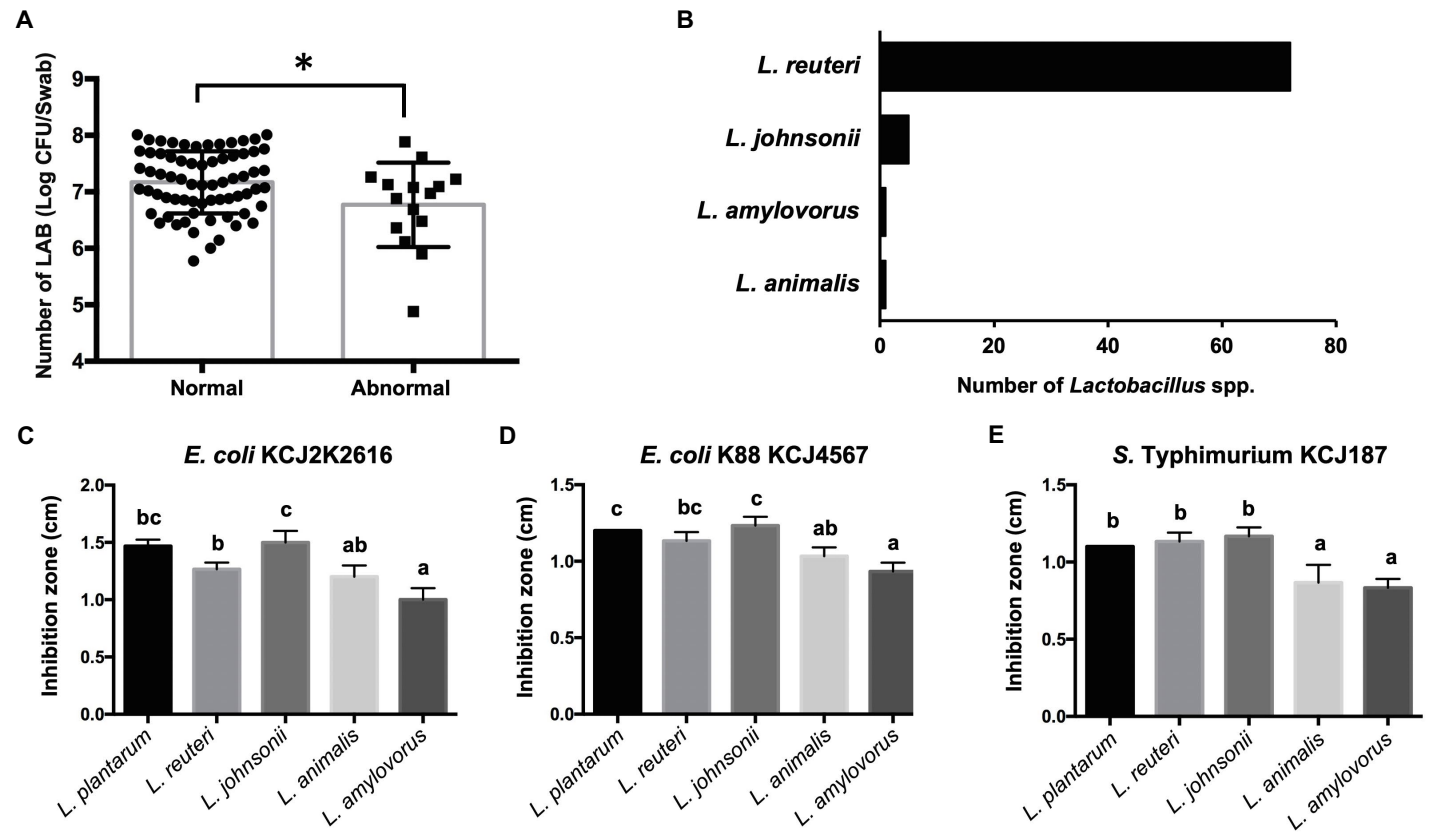
Isolation of *Lactobacillus* strains and their antimicrobial activity. **(A)** The concentrations of lactic acid bacteria (LAB) in normal and abnormal feces. In the bar graph, the asterisk reflects they are significantly different (*p*<0.05) based on the Student’s *t*-test. **(B)** Number of *Lactobacillus* spp. isolated from healthy calves identified by 16S rRNA gene sequencing. **(C-E)** Antimicrobial activity of *Lactobacillus* spp. against pathogenic strains. Bars with different letters indicate significant difference (*p*<0.05) based on one-way ANOVA followed by an *F*-test and Tukey’s HSD test for pairwise comparison of multiple means.

As we discovered higher abundance of *Lactobacillus* in normal feces (3.12%) compared to mild watery feces (2.02%) and severe watery feces (1.82%), as well as negative correlations between *Lactobacillus* and *Escherichia-Shigella* in normal feces, we isolated *Lactobacillus* strains and evaluated their antimicrobial activity against pathogens to test if these strains may contribute to the gastrointestinal tract health. The number of LAB in normal feces was significantly greater than that of abnormal feces (*p*=0.0198; Figure 6A), suggesting the concentrations of LAB might be associated with the gut health in the calves. In addition, 79 *Lactobacillus* spp. strains were isolated from the normal feces, and they were identified as *Limosilactobacillus reuteri* (formerly known as *Lactobacillus reuteri*), which was the most predominant species in the normal feces (Figure 6B). We also identified *Lactobacillus johnsonii*, *Lactobacillus amylovorus*, and *Ligilactobacillus animalis* (formerly known as *Lactobacillus animalis*) in the normal feces (Figure 6B). To test if these strains may suppress pathogenic strains as suggested in Figure 5, we conducted antimicrobial activity of these strains against *E. coli* and *S*. Typhimurium, which can cause calf diarrhea (Boranbayeva et al., 2020). All *Lactobacillus* spp. strains inhibited the growth of three pathogens (Figures 6C–E). Notably, *L. reuteri* and *L. johnsonii* showed clear inhibition that was similar to that of well-known probiotic strain *L. plantarum* against all test pathogens. *Lactobacillus reuteri* and *L. johnsonii* were the most and second most abundant *Lactobacillus* species in healthy calves (Figure 6B), suggesting these *Lactobacillus* spp. may affect gut health positively by suppressing the colonization of pathogenic bacteria in the gut of preweaning calves.

## Discussion

Diarrhea is a leading cause of death for beef and dairy calves in their first month of life (Caffarena et al., 2021). The homeostasis of gut microbial ecosystems is of central importance for maintaining the functions of the gastrointestinal tract of early preweaning calves (Virginio Junior and Bittar, 2021). However, there are limited studies reporting relationships between gut microbiota and diarrhea in young beef cattle. In this study, we investigated the fecal microbiota composition of one-month-old beef calves raised on pasture based on their fecal morphology. Many bacteria were identified to distinguish the normal solid feces and abnormal watery or hemorrhagic feces that had negative correlations with diarrheagenic pathogens.

The most apparent pattern for the abnormal fecal microbiota is the low bacterial richness. The number of bacterial types in feces was dramatically decreased with the severity of the watery or hemorrhagic phenomenon. A diverse gut microbiome is composed of 100 times more genes than host and serves as a functional expansion of host genomes (Zhu et al., 2010). The reduction in gut bacterial richness could result in limitation of microbiota function, including digestion, educating immune system, and inhibiting pathogens (Nicholson et al., 2012; Pickard et al., 2017; Schluter et al., 2020). The loss of microbial richness could be due to the great proliferation of pathogenic bacteria, such as *Fusobacterium* and *Campylobacter*. It is noteworthy that we found a significant lower birthweight of heifers with abnormal feces compared with those that had normal feces, suggesting the fetal development likely affects the gut microbiota maturation of young calves.

In this study, we screened for potential pathogenic and beneficial bacteria according to the fecal morphology. The bacteria enriched in abnormal feces include *Negativicutes*, *Tyzzerella*, *[Ruminococcus]_torques_group*, *Parasutterella*, *Veillonella*, *Lachnospiraceae*_UCG-004, *Lachnoclostridium*, *Butyricicoccus*, *Fusobacterium*, and *Campylobacter*. *Campylobacter*, including *C. jejuni*, *C. hyointestinalis*, and *C. fetus* are capable to cause irritation in the intestinal tract of calves and induce diarrhea (Firehammer and Myers, 1981; Diker et al., 1990). Previous studies reported that *Negativicutes*, *Veillonella*, and *Fusobacterium* were more abundant in diarrheic piglets compared to healthy weaned piglets (Huang et al., 2019; Karasova et al., 2021). In human studies, *Tyzzerella* contribute to neuroinflammation and its increase is associated with diarrhea (Nakajima et al., 2020). *Parasutterella* is in association with the development and progression of IBS (Chen et al., 2018). However, a few studies report the role of these bacteria in calf diarrhea.

We also identified several potential beneficial bacteria that showed higher relative abundance in normal feces, including *Christensenellaceae*, *Oscillospiraceae* (*Colidextribacter* and *Oscillospira*), *Barnesiella*, *Parabacteroides*, and *Lactobacillus*. Although many studies showed the beneficial effects of oral administration of *Lactobacillus* strains on reducing the diarrhea in swine and calves, only a few studies measured the change of *Lactobacillus* abundance between diarrheic and healthy beef cattle (Hou et al., 2015; Fernandez et al., 2020). In this study, we further isolated *Lactobacillus* strains from the healthy calves with normal feces, including *L. reuteri*, *L. johnsonii*, *L. amylovorus*, and *L. animalis*. In particular, the most abundant *Lactobacillus* species from healthy calves in our analysis, *L. reuteri* and *L. johnsnii*, are being administrated as potential probiotics and improved gastrointestinal health in pre-weaned calves (Fernandez et al., 2020). Jia et al. (2021) proposed *L. animalis* with high antibacterial activity against intestinal pathogens as anti-diarrheal probiotic for pig (Jia et al., 2021). Interestingly, the four *Lactobacillus* species isolated from the calves in this study all exerted antibacterial activities against pathogenic bacteria that commonly induce diarrhea in cattle, suggesting the high abundance of commensal *Lactobacillus* in healthy calves are beneficial in preventing colonization of pathogens.

In a previous study, Kim et al. (2021) applied FMT to effectively ameliorate calf diarrhea and found an increase in relative abundance in *Christensenellaceae* and *Parabacteroides* (Kim et al., 2021). Another study showed a significant decrease of *Oscillospira* in feedlot cattle with hemorrhagic diarrhea compared to healthy cattle (Zeineldin et al., 2018). Our study in line with other findings indicates the essential role of these bacteria in maintaining the cattle gut health. Although the function of *Barnesiella* in cattle is largely unknown, it showed impact in alleviating DSS-induced colitis in mice (Yang et al., 2020). Moreover, as we detected strong correlations between these potential beneficial bacteria with pathogenic bacteria, future studies will be directed to look into their competitive interactions.

## Data Availability Statement

The 16S rRNA gene amplicon sequencing data generated during the current study were submitted to NCBI under BioProject PRJNA751859.

## Ethics Statement

The animal study was reviewed and approved by University of Florida Institutional Animal Care and Use Committee.

## Author Contributions

PF and KJ designed the study. JD managed the animals. PF, GL, MK, YZ, and TL collected and processed the samples. PF, MK, and GL analyzed the data. PF, MK, GL, and KJ drafted the manuscript. PF, MK, and KJ finalized this manuscript. All authors contributed to the article and approved the submitted version.

## Funding

This study was funded by the Southern Sustainable Agriculture Research & Education Graduate Student Grant, GS19-206, supported by the National Institute of Food and Agriculture, U.S. Department of Agriculture to PF and KJ. MK was partly supported by Basic Research Program by the National Research Foundation of Korea, The Ministry of Education (2020R1A6A3A03038510).

## Conflict of Interest

The authors declare that the research was conducted in the absence of any commercial or financial relationships that could be construed as a potential conflict of interest.

## Publisher’s Note

All claims expressed in this article are solely those of the authors and do not necessarily represent those of their affiliated organizations, or those of the publisher, the editors and the reviewers. Any product that may be evaluated in this article, or claim that may be made by its manufacturer, is not guaranteed or endorsed by the publisher.

## Footnotes

^1^ https://www.arb-silva.de/documentation/release-1381/

^2^ http://gephi.org
