# Supplementary material for: The Gut Microbiota of Newborn Calves and Influence of Potential Probiotics on Reducing Diarrheic Disease by Inhibition of Pathogen Colonization
Source: Front Microbiol. 2021 Oct 21;12:772863. doi: 10.3389/fmicb.2021.772863 (PMC8567051; doi:10.3389/fmicb.2021.772863)
Supplement: Supplementary file 1 [file Data_Sheet_1.zip › Supplementary figure.pdf]

Supplementary information for

The gut microbiota of newborn calves and influence of potential probiotics on reducing diarrheic disease by inhibition of pathogen colonization

Peixin Fan[1, 2, ¶], Miju Kim[1,2, ¶], Grace Liu[1,2], Yuting Zhai[1,2], Ting Liu[1,2], J. Danny Driver[2], and Kwangcheol C. Jeong[1, 2*]

[1]Emerging Pathogens Institute, University of Florida, Gainesville, FL 32611 USA

[2]Department of Animal Sciences, University of Florida, Gainesville, FL 32611 USA

Running Title: Gut microbiota of preweaned beef cattle

* correspondence to Kwangcheol C. Jeong, kcjeong@ufl.edu

¶ These authors contributed this paper equally.

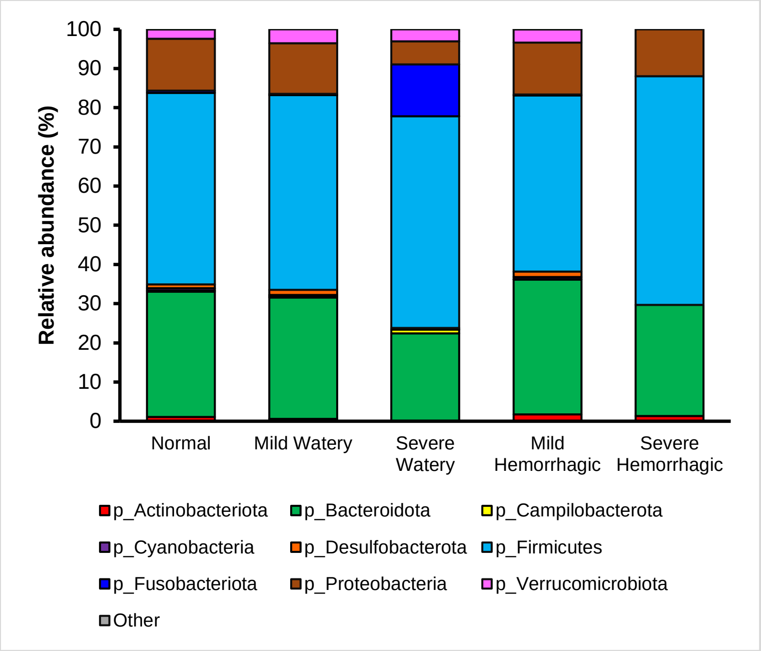

Supplementary Figure 1 Distribution of abundant bacterial phyla in feces with different morphologies.
